# Promoting Adolescent and Youth Health Through Physical Activity Initiatives and Interventions in Sub-Saharan Africa: The ARISE-NUTRINT and DASH Initiatives

**DOI:** 10.3389/ijph.2025.1608609

**Published:** 2025-09-29

**Authors:** Nikola Todorovic, Marijana Ranisavljev, Darinka Korovljev, Joy Mauti, Christine Neumann, Innocent Mboya, Elisabetta Ferrero, Millogo Ourohiré, Sylvain Somé, Shuyan Liu, Sachin Shinde, Ramadhani A. Noor, Till Bärnighausen, Sergej M. Ostojic

**Affiliations:** ^1^ Public Health Nutrition Division, Center for Health, Exercise and Sport Sciences, Belgrade, Serbia; ^2^ Faculty of Sport and Physical Education, University of Agder, Kristiansand, Norway; ^3^ Heidelberg Institute of Global Health, Heidelberg University Hospital, Heidelberg, Baden-Württemberg, Germany; ^4^ Africa Academy for Public Health, Dar es Salaam, Tanzania; ^5^ Department of Epidemiology and Biostatistics, School of Public Health, KCMC University, Moshi, Tanzania; ^6^ Department of Global Health and Population, Harvard T.H. Chan School of Public Health, Boston, MA, United States; ^7^ Health Sciences Research Institute, Ouagadougou, Burkina Faso; ^8^ Nouna Health Research Center, Nouna, Burkina Faso; ^9^ Department of Psychiatry and Psychotherapy (Campus Charité MiIe), Charité -Universitätsmedizin Berlin, Berlin, Germany; ^10^ German Center for Mental Health (DZPG), Berlin and Heidelberg, Germany; ^11^ Center for Inquiry into Mental Health, Pune, Maharashtra, India; ^12^ Department of Nutrition and Public Health, University of Agder, Kristiansand, Norway

**Keywords:** youth, physical activity, Sub-Saharan Africa, non-communicable diseases, ARISE-NUTRINT

## Abstract

Regular physical activity (PA) is essential for maintaining health and wellbeing across all life stages, particularly in children and adolescents. Despite its benefits, most adolescents fail to meet the World Health Organization’s PA recommendations, with global trends indicating alarmingly low participation rates, particularly among girls. This issue is pronounced in Sub-Saharan Africa (SSA), where physical inactivity is a significant public health concern, contributing to rising obesity rates. Environmental, socio-economic, and cultural barriers further exacerbate low PA engagement, including extreme weather conditions, lack of recreational infrastructure, gender norms, and economic constraints. Addressing these challenges requires targeted interventions, including education, improved infrastructure, and policy implementation. Innovative programs such as ARISE-NUTRINT and DASH aim to enhance adolescent health in SSA through nutrition and PA-focused strategies. This commentary paper explores existing barriers to PA, evaluates promising on-going efforts and interventions, and highlights opportunities for promoting PA in SSA through community engagement, digital platforms, and cross-sector collaboration. Implementing sustainable and culturally tailored strategies is crucial to reversing current trends and fostering long-term health benefits for adolescents in SSA.

## Background

Regular physical activity (PA) is critical for maintaining physical, mental, and social health at all stages of life. It helps to prevent and manage non-communicable diseases (NCDs), improving mental health and quality of life. PA is particularly important in children and adolescents for supporting healthy growth, promoting optimal bone density, enhancing cognitive function, and fostering overall wellbeing [1]. Despite these benefits, recent data indicates that the majority of adolescents do not meet the recommendation from the World Health Organization (WHO) regarding PA guidelines [2]. For example, an analysis of data in 2016 from 146 countries, territories, and regions encompassing a sample of 1.6 million adolescents aged 11–17 indicated that 81% of this population had insufficient levels of PA [3]. Notably, the prevalence of insufficient PA was higher in females (84.7%) than in males (77.6%) [3].

Similar trends are observed in Sub-Saharan Africa (SSA), where physical inactivity ranks among the top ten risk factors contributing to the disease burden [4]. SSA is gaining increased attention due to its rapidly growing population and improving sociodemographic and economic conditions. With approximately a quarter of its population aged 10–24 years, SSA is experiencing a rapid rise in overweight, affecting 10%–25% of adolescents in the region, with girls being 1.5 to 2 times likely to be affected than boys [5]. Increased physical activity, combined with better diets and supportive environments, can reverse the trend, and hence a critical need for inclusive public health initiatives focused on promoting PA among adolescents and youths, especially prioritizing girls in this region. A recent study assessing PA levels among 64,127 in-school adolescents aged 12–17 years from 23 African countries found that only 20% met the WHO’s recommended PA levels [6]. When analyzed by gender, 25% of male adolescents and 16% of female adolescents achieved sufficient activity. PA levels varied significantly by country, with Sudan showing the lowest rate at 11.6% and Benin the highest at 38.3%. Overall, only one in five adolescents in the surveyed countries met the WHO recommendations for PA.

More than half of adults and a third of children and adolescents are globally predicted to be overweight or obese by 2050, and rates increasing exponentially in SSA [7]. In the past three decades, Africa and Asia have observed the largest percentage increases in obesity prevalence in the world. Populated SSA countries like Nigeria already stand out worldwide with its predicted rise in adults with overweight and obesity, with the number projected to more than triple from 36.6 million in 2021 to 141 million in 2050—making it the country with the fourth-largest population of adults with overweight and obesity [7]. The growing population and rapid urbanization of the African continent is projected to exacerbate these trends by reducing access to sports facilities and spaces for PA, highlighting the urgent need for robust initiatives and interventions to counter the low levels of PA, and prevent a complete transition to obesity for children and adolescents in SSA [7]. However, a better understanding of “which interventions work and how” to improve physical activity among adolescents in the specific African context remains a significant gap. This paper explores the challenges and barriers contributing to this issue, as well as the opportunities and strategies for promoting PA, aiming to provide a brief understanding of the current situation and provide practical solutions and actions.

## Challenges and Barriers

**FIGURE 1:**
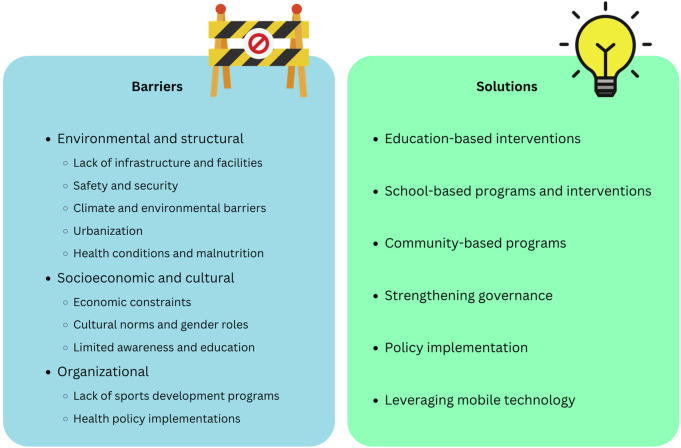
Challenges and opportunities for the development of physical activity programs in Sub-Saharan Africa (Belgrade, Serbia, 2020).

Several strategies have been proposed to enhance PA in the SSA region [8, 9]. One such initiative, Nyakaza-Move-for-Health, followed a structured six-step framework: (a) Needs assessment, (b) Development of a logic model of the problem (LMP), (c) Formulation of program outcomes and objectives, (d) Program design and production, (e) Implementation planning, and (f) Intervention evaluation planning [9]. In a separate randomized controlled trial conducted in South Africa [8], the authors assessed the impact of a 12-month health-promotion intervention on physical activity levels and behavioral change among university students. A total of 171 participants were evaluated using self-reported physical activity measures. The results indicated a general increase in physical activity, accompanied by positive changes in other lifestyle behaviors. However, significant barriers remain, emphasizing the intervention’s effectiveness while underscoring the need for sustained, long-term strategies. These barriers can be categorized into three main groups: environmental and structural barriers, socio-economic and cultural factors and organizational barriers (Figure 1). Still, the findings from the above studies are based on data from South Africa and may not be representative of the broader SSA region; future initiatives should aim to identify which barriers are context-specific and may not be generalizable across the diverse populations of SSA.

Environmental barriers significantly hinder the development of PA programs in SSA. Extreme weather conditions, such as high temperatures and droughts, often render outdoor activities impractical. In urban areas, elevated air pollution, personal safety fears, parks and gym facility vandalism, and diminishing green spaces further restrict safe PA opportunities [10]. Beyond environmental challenges, insufficient investment in recreational sports programs compounds the issue. Despite the potential of sports to enhance health, inclusivity, and social cohesion, they remain poorly integrated into education and health policies. Organized activities that could foster PA among adolescents receive little support, particularly in rural and low-income urban areas, further restricting participation [11]. Additionally, inadequate infrastructure exacerbates these barriers. Many regions lack accessible parks, recreational centers, and sports facilities, while schools are often underfunded and unable to provide quality physical education or extracurricular activities. Poor maintenance of existing facilities further discourages participation, particularly among low-income and rural communities [12]. Poor health conditions and malnutrition present additional obstacles, where many children and adolescents in SSA suffer from undernutrition, anemia, and other diet-related deficiencies, impairing physical performance and motivation for exercise [13].

Socio-economic and cultural factors further limit PA engagement. Traditional gender roles in many SSA communities discourage female participation, as household responsibilities take precedence and sports are often perceived as male-dominated [14]. Limited awareness of PA’s health benefits exacerbates these issues, as inadequate health promotion and the absence of PA education in schools leave many unaware of its role in preventing chronic diseases.

Economic constraints present another significant barrier. High poverty levels across SSA make sports participation unaffordable, with costs for equipment, gym memberships, and club fees often prohibitive. Limited government funding for PA infrastructure and programs shifts the financial burden onto individuals. Additionally, in many low-income households, leisure activities are deprioritized as individuals focus on income-generating work [15].

A critical issue in the Sub-Saharan African context is the relative scarcity of programs specifically tailored to the needs of adolescents, particularly girls. While several initiatives—such as school-based physical education programs, community-led campaigns, and broader national policy frameworks—have been implemented to promote physical activity among children and adolescents, these efforts often vary in reach, consistency, and effectiveness across different regions. Many school-based programs are constrained by limited resources, lack of trained personnel, and inadequate infrastructure, which restrict their capacity to deliver regular and engaging physical activity. Similarly, while community-driven campaigns have shown promise in raising awareness and encouraging participation, they are frequently short-term, underfunded, or fail to address the unique socio-cultural barriers that limit adolescent, especially female, engagement in structured physical activity. National policy initiatives, though commendable in their scope, often suffer from limited implementation at the local level and insufficient integration with educational and health systems. Consequently, women, adolescents, and young children remain underserved in many areas due to the limited adaptability and sustainability of current interventions. Addressing these challenges will require not only scaling up and better resourcing of existing programs but also the development of context-specific, age-appropriate, and gender-sensitive interventions that promote long-term engagement in physical activity and foster the acquisition of physical, psychological, and social benefits.

Finally, although each country has its own health policies, most SSA countries adopt global health strategies [16]. However, effective policy transfer requires local adaptation, stakeholder engagement, adequate resources, and strong inter-organizational collaboration. Addressing these challenges demands targeted interventions focused on infrastructure investment, culturally sensitive education, economic support, and environmental adaptations—key measures for combating physical inactivity and advancing health and social progress in SSA.

## Possibilities and Opportunities

While numerous challenges persist, there is substantial potential for advancements and innovative interventions in promoting PA in SSA. A key strategy is fostering active societies by increasing awareness of PA benefits for all demographics, encompassing various age groups—not limited to adolescents, and equally among men and women. Prior interventions incorporating educational and media-based approaches have effectively increased adolescent PA levels, with positive changes sustained over a 6-month follow-up period [9].

PA is a low-cost and widely accessible intervention, yet its impact in the SSA region remains limited. Existing policies and action plans aimed at promoting PA are often only partially implemented. Educating adolescents in SSA about the benefits of PA is crucial, as habits formed during this stage can impact lifelong health. Schools in this region play a vital role, where integrating health and physical education can raise awareness about how regular activity improves mental wellbeing, academic performance, and physical health [17]. In addition, community-based programs and partnerships are necessary to provide structured, accessible, and diverse activities—such as team sports, recreational activities, and fitness options—ensuring that PA is both affordable and engaging [18]. Additionally, utilizing mobile technology and digital platforms among adolescents in SSA, can help promote health messages in a relatable and effective way, reinforcing positive behaviors and encouraging long-term PA engagement [19].

Improving urban infrastructure to create supportive environments is essential for making PA accessible and encouraging greater participation [20]. Expanding safe, well-maintained spaces like parks, walking paths, and sports facilities allows people of all demographics and settings to engage in regular PA, which is linked to numerous physical and mental health benefits [21]. Providing varied opportunities, such as community sports programs, exercise classes, and school-based activities, further helps integrate movement into daily routines and fosters lasting healthy habits [22]. Effective governance and management, including clear leadership and well-organized structures, are also critical. Building partnerships between government, health organizations, schools and community structures ensures that efforts to promote PA are consistent and widely supported [23]. Creating practical, easy-to-implement solutions—such as policies supporting active transportation, school-based fitness programs, and community-wide events—holds strong potential to improve PA levels across communities, promoting long-term health benefits [24].

While we outline several opportunities for promoting PA, it is important to acknowledge that not all barriers identified—particularly those related to cultural norms and gender roles—are fully addressed by the proposed solutions. Cultural perceptions that prioritize domestic responsibilities for girls, or that frame sports as male-dominated activities, continue to limit PA participation among female adolescents in many SSA communities. Addressing these deeply rooted social norms requires culturally sensitive interventions that engage families, schools, and community leaders to reshape attitudes toward gender and PA. Programs should promote inclusive messaging, provide female role models in sport and health promotion, and offer safe, gender-appropriate spaces for girls to participate in PA. Integrating these strategies into broader community and policy initiatives will be essential to overcoming persistent cultural barriers and ensuring equitable access to PA opportunities for all adolescents.

## ARISE-NUTRINT and DASH Initiatives

To tackle pressing public health challenges, collaborators from SSA, Europe, and North America established the multidisciplinary ARISE-NUTRINT initiative (Africa Research, Implementation Science, and Education – Reducing NUTRition-related non-communicable diseases in adolescence and youth: INTerventions and policies to boost nutrition fluency and diet quality in Africa), funded by the European Union [25]. The initiative has already made significant progress by implementing nutrition-based interventions and formulating ongoing plans for practical, region-specific solutions [26]. Furthermore, the DASH (Research Network for Design and Evaluation of Adolescent Health Interventions and Policies in Sub-Saharan Africa) initiative, funded by the German Federal Ministry of Education and Research (BMBF), focuses on adolescent health, in particular on healthy nutrition and physical activity in addition to mental health and violence and sexual and reproductive health. However, malnutrition and obesity are inherently complex and interconnected issues, often influenced by a combination of nutritional, behavioral, and environmental factors. To address these challenges comprehensively, the project must also incorporate strategies to combat the growing problem of physical inactivity in this region.

Several actions are planned to address these challenges, starting with raising awareness about the consequences of physical inactivity and developing educational strategies to encourage healthier behaviors. A key initial step involves conducting a comprehensive scoping review to evaluate existing knowledge and identify gaps, forming the foundation for targeted interventions. The scoping reviews will be followed by the application of human-centered design to co-design and validate the context specific PA interventions among adolescents and youths in SSA. Based on the findings, the initiative plans to implement simple, cost-effective interventions tailored specifically to the needs and possibilities of adolescents in SSA. These interventions will aim to promote PA through practical strategies that are accessible, culturally relevant, and sustainable in the long term. Achieving these goals requires a multidisciplinary approach, with collaboration across health, education, and community sectors to prioritize PA awareness as a key component of healthier lifestyles for adolescents in SSA.

### Conclusion

Physical inactivity among adolescents in Sub-Saharan Africa presents a pressing and multifaceted public health challenge, contributing to rising rates of overweight, obesity, and associated non-communicable diseases in the region. Despite the well-established benefits of regular physical activity for physical, cognitive, and psychosocial development, the majority of adolescents—especially girls—fail to meet recommended activity levels. A combination of environmental, socioeconomic, cultural, and policy-related barriers impedes progress, highlighting the need for tailored, context-specific solutions. However, this challenge also offers significant opportunities. School-based education, community-driven programs, improved infrastructure, and the use of digital tools can promote engagement and behavior change. Initiatives such as ARISE-NUTRINT and DASH demonstrate promising steps toward integrated, evidence-based interventions. Moving forward, a coordinated, multisectoral approach is essential to implement inclusive, scalable, and sustainable physical activity strategies that prioritize adolescents’ needs—especially girls and marginalized populations—across diverse settings in Sub-Saharan Africa. Through such efforts, physical activity can be elevated as a cornerstone of youth health and development in the region.
